# Ten Years’ Experience Using Proxymetacaine Hydrochloride 0.5% for Postoperative Pain Control in Epithelium-Off Corneal Crosslinking †

**DOI:** 10.3390/jcm14134692

**Published:** 2025-07-02

**Authors:** Mohamed Gamal Elghobaier, Issac Levy, Mayank A. Nanavaty

**Affiliations:** 1 Sussex Eye Hospital, University Hospitals Sussex NHS Foundation Trust, Eastern Road, Brighton BN2 5BF, UK; 2 Ophthalmology Department, Rabin Medical Center, Petach Tikva 4941492, Israel; 3 Faculty of Medicine, Tel Aviv University, Tel Aviv 6997801, Israel; 4 Brighton & Sussex Medical School, University of Sussex, Falmer, Brighton BN1 9PX, UK

**Keywords:** crosslinking, epithelium-off, postoperative pain, anaesthetic

## Abstract

**Background/Objectives**: To evaluate the efficacy and safety of using the preservative-free topical proxymetacaine hydrochloride (Minims, 0.5% *w*/*v*, Bausch & Lomb, UK) to control postoperative pain after epithelium-off corneal crosslinking (CXL) for keratoconus. **Methods**: This is an observational study of patients with mild to severe keratoconus who have undergone epithelium-off CXL. CXL was completed by applying dextran-free riboflavin (0.1%) for 10 min (Vibex Rapid; Avedro, Inc.), followed by continuous UV-A light (Avedro KXL system; Avedro, Inc.) for 30 min at an intensity of 3 mW/cm^2^ and an energy of 5.4 J/cm^2^. All patients were prescribed postoperative proxymetacaine hydrochloride PRN with an allowed frequency of up to eight times per 24 h for the first 3 days to control postoperative pain. Patients were reviewed at 1–2 weeks postoperatively for a comprehensive examination, including assessment of delayed corneal healing, removal of the bandage contact lens, and recording of subjective symptoms. **Results**: There were 223 eyes of 180 patients with a mean age of 24.9 ± 8.6 years (range: 13–38 years). Male patients were 72%. At their planned first postoperative visit, we found no corneal healing abnormalities, such as persistent epithelial defects, epithelial irregularities, or early postoperative stromal haze, in any patient. All patients subjectively reported that proxymetacaine drops helped them to control postoperative pain, particularly in the first 48 h. **Conclusions**: None of the patients reported pain after 3 days of using proxymetacaine drops up to eight times a day for the first 3 days. It appears to be a safe and effective solution to control postoperative pain without any complications.

## 1. Introduction

When corneal crosslinking (CXL) was initially introduced in 1998, the original Dresden protocol involved removing the epithelium, followed by the application of riboflavin (Vitamin B2) eye drops, and subsequently treating the patient with ultraviolet-A (UVA) light [1,2]. The most well-known technique for CXL is the epithelium-off, or epi-off, procedure [3,4].

Epithelium-off (epi-off) corneal crosslinking has consistently demonstrated superior efficacy compared to alternative crosslinking modalities in the treatment of progressive keratoconus and corneal ectasia, establishing itself as the gold standard for halting disease progression [3,5,6]. The fundamental advantage of epi-off crosslinking stems from the complete removal of the corneal epithelium, which eliminates the natural barrier that significantly impedes riboflavin penetration and ultraviolet-A (UV-A) light transmission into the corneal stroma [3,6]. Clinical evidence overwhelmingly supports the superior therapeutic outcomes of epi-off crosslinking, with studies demonstrating significantly greater topographic flattening effects compared to epithelium-on (epi-on) procedures, achieving corneal flattening of approximately 2.0 diopters versus only 0.45 diopters with epi-on techniques [6]. Meta-analyses comparing epi-off and epi-on crosslinking have revealed that while both techniques can stabilise keratoconus progression, epi-off crosslinking provides more consistent and predictable outcomes, with randomised controlled trials showing that maximum keratometry (Kmax) values decreased significantly in the epi-off group but paradoxically increased in the epi-on group at 36-month follow-up [7]. The enhanced efficacy of epi-off crosslinking is further evidenced by deeper demarcation line formation, typically reaching 320 μ compared to only 190 μ with transepithelial approaches, indicating more extensive stromal crosslinking and biomechanical strengthening [7]. While epi-on crosslinking techniques, including iontophoresis-assisted transepithelial crosslinking, have been developed to reduce postoperative complications and patient discomfort [8,9], these modifications come at the cost of reduced therapeutic efficacy, with studies reporting progression rates of 20% after iontophoresis-assisted crosslinking compared to only 7.5% after standard epi-off crosslinking [10]. The superiority of epi-off crosslinking extends beyond keratometric improvements to include better visual outcomes, with accelerated epi-off protocols demonstrating comparable efficacy to standard Dresden protocol while maintaining the fundamental advantages of epithelial removal [11,12]. Recent comprehensive evaluations have confirmed that despite the higher incidence of postoperative complications such as delayed epithelial healing and stromal haze associated with epi-off procedures, the enhanced biomechanical strengthening and consistent halting of disease progression justify its continued use as the preferred treatment modality [13]. The anatomical rationale for epi-off superiority is well-established, as the intact epithelium and Bowman’s layer can block approximately 30% of UV-A light energy and prevent adequate riboflavin saturation of the stromal tissue, leading to suboptimal crosslinking effects and potential treatment failure. Long-term follow-up studies spanning up to five years have consistently demonstrated that epi-off crosslinking provides durable stabilisation of corneal ectasia with minimal risk of disease progression. In contrast, alternative techniques show higher rates of breakthrough progression and require closer monitoring [9]. Consequently, while emerging crosslinking modalities continue to be investigated for their potential to reduce patient morbidity, epi-off crosslinking remains the most effective intervention for preventing keratoconus progression and delaying the need for corneal transplantation. This epi-off technique has several drawbacks, including a risk of infection, scarring, and epithelial healing defects. However, the main concern for patients is postoperative pain [14,15,16,17]. The corneal epithelial cells, characterised by their tight junctions, are removed to enhance riboflavin penetration into the cornea stroma. Epithelial defects usually last for a few days; subsequently, patients experience pain in the first 24–48 h postoperatively [18]. The size of the corneal epithelial defect only partially accounts for the level of postoperative pain, which is likely affected by other unknown factors [19]. This poses particular challenges for paediatric and developmentally delayed groups, who are increasingly experiencing CXL [20].

Different perioperative and postoperative approaches have been explored to attempt pain reduction after CXL, but no method is universally accepted [19,20,21,22,23]. Proxymetacaine hydrochloride 0.5% is a widely used topical anaesthetic due to its safety, efficacy, and high tolerability compared to other topical anaesthetics employed for ophthalmic pain management [24,25]. The use of proxymetacaine hydrochloride 0.5% in epi-off CXL has not been explored. This study aims to demonstrate the long-term efficacy and safety of using preservative-free topical anaesthetic drops, proxymetacaine hydrochloride (Minims, 0.5% *w*/*v*, Bausch & Lomb, Kingston, UK), for controlling postoperative pain associated with epi-off corneal crosslinking (CXL) in patients with keratoconus.

## 2. Patients and Methods

This study was designed as a single-centre, observational, consecutive case series. It was approved by the Clinical Audit Committee at Sussex Eye Hospital, University Hospitals Sussex NHS Foundation Trust (audit registration number: 2129). The study was conducted in accordance with the principles outlined in the Declaration of Helsinki. As part of routine clinical practice, informed consent was obtained from the patients to collect data for audit purposes before surgery.

Progressive keratoconus was diagnosed based on clinical and tomographic features. It was defined as one or more of the following changes over 12 months: an increase of 1.00 D or more in the steepest keratometry measurement, an increase of 1.00 D or more in the manifest cylinder, or an increase of 0.50 D or more in manifest refraction spherical equivalent (MRSE) [26,27,28]. The inclusion criteria were patients of any age with a diagnosis of progressive keratoconus who had corneal CXL between January 2014 and January 2024. Exclusion criteria were history of acute hydrops, visible corneal scarring or oedema on slit-lamp examination, excessive eye rubbing, severe atopy, ocular surgery or trauma, corneal thinnest pachymetry ≤400 μm, and iatrogenic ectasia or other ectasias like pellucid marginal degeneration. Differentiation between pellucid marginal degeneration and peripheral keratoconus was a clinical decision based on the patient’s age at presentation, topographic features, the axis of astigmatism, high or low prolate shape of the cornea, and wavefront aberrations.

The crosslinking protocols employed are described below. In brief, under aseptic conditions, proxymetacaine hydrochloride 0.5% minims (Bausch & Lomb, UK) and tetracaine hydrochloride 0.5% minims (Bausch & Lomb, UK) were applied for topical anaesthesia. A speculum was inserted, and the central 9.0 mm of the cornea was exposed to 20% ethanol solution for 30 s, followed by the mechanical removal of the epithelium using a sponge spear (Sterile PVA (polyvinyl alcohol) Foam Sponge). The stroma was soaked in riboflavin 0.1% (Vibex Rapid, Avedro, Burlington, MA, USA) for 10 min at 2 min intervals. This was followed by continuous UV-A light exposure (Avedro KXL system, Waltham, MA, USA) at an intensity of 3.0 mW/cm^2^ and a total energy dose of 5.4 J/cm^2^ for 30 min (Table 1). At the end of the procedure, the corneal surface was washed out, and a bandage contact lens (BCL) was inserted. Patients were prescribed Ofloxacin 0.3% (EXOCIN^®^ (Allergan Inc., Irvine, CA, USA) 3 mg/mL) and prednisolone acetate 1% (Pred Forte, Allergan, Westport, Ireland) four times a day for 14 days along with G. Proxymetacaine Hydrochloride 0.5% (Bausch & Lomb Minims, Rochester, NY, USA) minims to be used up to 8 times a day for first 3 days only. The patients were instructed to use proxymetacaine hydrochloride 0.5% as infrequently as possible, but not to exceed 8 times a day and for the first three days only. Only one box of proxymetacaine hydrochloride 0.5% containing only 20 minims was given to the patients. Between the fourth and seventh postoperative days, we contacted all patients to inquire about any symptoms of pain or discomfort they may have experienced. Any pain beyond three days postoperatively was documented. Then, patients were seen between one and two weeks following the procedure, and the BCL was removed at this stage. An objective assessment of the cornea was performed using the slit lamp, and fluorescein staining was employed to evaluate the ocular surface. As per our departmental protocol, all crosslinking patients were followed up at 3, 6, 12, and 6 months thereafter for regular objective tomography and slit-lamp assessment.

Proxymetacaine hydrochloride 0.5% is a rapidly acting topical ophthalmic anaesthetic widely utilised in clinical practice for its efficacy in providing transient corneal and conjunctival anaesthesia during diagnostic and minor surgical procedures. As an aminoester derivative, proxymetacaine primarily functions by antagonising voltage-gated sodium channels, stabilising neuronal membranes, and inhibiting the initiation and propagation of action potentials in sensory nerve endings. This mechanism results in rapid onset of anaesthesia, typically within 30 s of instillation, with clinically effective numbness persisting for 15–20 min under standard dosing regimens. The drug’s pharmacokinetic profile demonstrates rapid corneal penetration due to its lipophilic properties; however, systemic absorption remains minimal when used appropriately, thereby minimising the risks of systemic toxicity. Clinical applications include the use during tonometry, foreign body removal, suture extraction, and preoperative anaesthesia for ocular surgeries, with studies demonstrating enhanced efficacy when combined with cycloplegic agents like cyclopentolate hydrochloride. Research by Doyle et al. revealed that proxymetacaine pre-treatment significantly accelerates cyclopentolate absorption, enabling accurate autorefraction measurements within 10 min compared to 20 min without anaesthetic pre-treatment, thereby optimising clinical workflow in paediatric populations [29]. This synergism arises from proxymetacaine’s epithelial disruption, which facilitates deeper drug penetration while simultaneously improving patient comfort during instillation [29]. The safety profile of proxymetacaine 0.5% remains favourable when used judiciously, though its potential for ocular surface toxicity necessitates strict adherence to recommended dosing limits. Transient side effects include mild conjunctival hyperaemia (15–20% of patients), transient stinging upon instillation (30–40%), and rare instances of allergic conjunctivitis (<2%). However, prolonged or frequent use beyond the maximum recommended 5–7 doses per episode can precipitate toxic keratopathy, characterised by persistent epithelial defects, stromal infiltrates, and, in severe cases, corneal melting [30]. Histopathological studies demonstrate proxymetacaine’s direct cytotoxic effects on corneal epithelial cells, including disruption of actin filaments, inhibition of cell migration, and induction of apoptosis at concentrations exceeding therapeutic levels [30,31]. Comparative toxicology assessments reveal proxymetacaine exhibits four-fold greater in vitro cytotoxicity than cocaine and equivalent epithelial toxicity to tetracaine at equimolar concentrations, underscoring the importance of dosage control [31]. Clinical case series document bilateral corneal calcific infiltrates and neurotrophic ulcers in patients self-medicating with proxymetacaine for 7–50 days, often requiring surgical interventions such as amniotic membrane transplantation or penetrating keratoplasty [32,33]. These complications arise from the drug’s dual inhibition of epithelial healing mechanisms and nociceptive signalling, creating a vicious cycle of pain–anaesthetic abuse–tissue damage [30,32,33]. Recent pharmacodynamic investigations using non-contact corneal aesthesiometry challenge traditional duration estimates, demonstrating that proxymetacaine’s anaesthetic effects persist beyond 60 min post-instillation, with maximum depth achieved at 15 min [34]. This prolonged activity, while beneficial for extended procedures, increases addiction potential in susceptible individuals. Management of toxic keratopathy mandates immediate cessation of anaesthetic use, substitution with oral analgesics, and aggressive lubrication with preservative-free artificial tears [30,32,33]. Prophylactic measures include educating prescribers about the drug’s abuse potential and restricting access to single-dose units [32,33]. Despite these risks, proxymetacaine remains indispensable in ophthalmic practice due to its rapid onset and superior patient tolerance compared to alternatives such as tetracaine, which induces greater initial irritation [31]. Ongoing research explores reformulations with reduced preservative content (notably benzalkonium chloride) to mitigate epithelial toxicity while maintaining anaesthetic efficacy [30].

A retrospective chart review of patients who underwent surgery between January 2014 and January 2024 was performed. Our hospital used standardised medical records across all our hospital sites. All data were extracted from these standardised hospital medical records that have been consistently utilised across our institution throughout the ten-year study period. Every encounter between medical staff and the patient, whether in person or remotely, is documented in the patient’s medical record. This ensured the completeness, consistency, and reliability of clinical parameter documentation for retrospective analysis. An Excel spreadsheet (Microsoft Corp., USA) was used for data analysis, from which the mean and standard deviation were calculated for continuous data and as a percentage for categorical data. The primary outcome was to evaluate the number of patients reporting pain beyond three days. Secondary outcomes included persistent epithelial defect at the first postoperative visit, which occurred between one and two weeks after the procedure, any additional interventions required to aid non-healing epithelial defect, as well as other adverse events, and whether age or gender influenced subjective pain management.

## 3. Results

We identified 223 eyes from 180 patients who underwent epi-off CXL over ten years. The study cohort had an average age of 24.9 ± 8.6 years, ranging from 13 to 38 years. Seventy-two percent of the total participants were male.

No patients in this study reported experiencing pain lasting more than three days following the procedure. None of the patients were identified with persistent epithelial defects between 7 and 10 days after the procedure; subsequently, none required any additional procedure interventions to aid non-healing epithelial defects, and none had any adverse events. Basic descriptive statistics were used, and no significant correlations were found between subjective pain control and age or sex.

## 4. Discussion

The objective of our study was to identify a solution for the postoperative pain experienced by all patients undergoing epi-off corneal collagen crosslinking (CXL). For the past decade, we have used proxymetacaine hydrochloride 0.5% as a standard postoperative protocol for all our patients, achieving effective pain control without any complications.

Topical anaesthetics are critical in ophthalmology for pain-free diagnostics and surgeries. Proxymetacaine (proparacaine hydrochloride 0.5%) stands out due to its rapid onset, minimal discomfort, and favourable safety profile [35,36]. There are studies comparing proxymetacaine with tetracaine, lidocaine, and oxybuprocaine, focusing on pharmacological properties, clinical efficacy, safety, and emerging innovations [31,37]. Proxymetacaine, an aminoester anaesthetic, blocks voltage-gated sodium channels in corneal nerves. Its ester structure allows hydrolysis by plasma cholinesterase, reducing systemic accumulation compared to aminoamides like lidocaine [31,36]. Tetracaine shares this mechanism. Still, higher lipid solubility prolongs corneal contact [31,38]. Proxymetacaine acts within 30 s, peaking at 15 min, with effects lasting 10–15 min—sufficient for tonometry or removal of foreign bodies [35,39]. Tetracaine’s onset is slower (1–2 min), but the duration extends to 20–30 min, which is beneficial in photorefractive keratectomy (PRK) [40]. Lidocaine 2% gel bridges this gap, offering a 2–3 min onset and 30–45 min of anaesthesia, ideal for paediatric strabismus surgery [41,42].

In tonometry, proxymetacaine’s rapid action minimises workflow disruption. A crossover trial (*n* = 40) showed that rebound tonometry under proxymetacaine caused a negligible IOP reduction (−1.6 mmHg vs. benoxinate’s −0.8 mmHg), thereby preserving accuracy [43]. Tetracaine’s prolonged effect aids serial measurements but risks artefacts from epithelial exposure. For cataract surgery, the painless instillation of proxymetacaine is preferred. A trial (*n* = 40) found equivalent intraoperative analgesia to tetracaine but lower instillation pain (0 vs. 24 mm VAS) [38]. Conversely, PRK patients reported 1.5-point lower pain scores at 30 min with tetracaine, highlighting its duration advantage [40]. Lidocaine gel reduces postoperative ibuprofen use by 53% compared to proxymetacaine in strabismus surgery (*n* = 140), attributed to its prolonged contact [42]. However, proxymetacaine remains safer for brief procedures like suture removal due to its lower contamination risk [42,44]. All topical aesthetics inhibit epithelial healing. Proxymetacaine delays rabbit corneal healing by 18 h, compared to tetracaine’s 24 h [31,36]. Abuse cases report severe keratopathy with calcific infiltrates and ulcers requiring keratoplasty [37,44]. Lidocaine gel poses a 19.4% risk of punctate keratitis post-intravitreal injection due to residue [41,42]. Proxymetacaine’s metabolism minimises systemic toxicity, with no cardiovascular events in a 556-patient meta-analysis [37]. Tetracaine carries higher allergy risks, including anaphylactoid reactions at doses >20 mg [31,44]. Lidocaine gel requires caution in cardiac patients due to absorption risks [41,42]. Combining proxymetacaine with tetrodotoxin (TTX) extends anaesthesia to 60 min while halving the proxymetacaine dose in preclinical studies [45]. Octyltrimethylammonium bromide (OTAB) enhances efficacy, enabling lower-concentration formulations [36,45]. Nanoparticle-encapsulated proxymetacaine maintains therapeutic levels for 8 h post-instillation, promising for postoperative pain management in refractive surgery [36,45]. So, proxymetacaine excels in rapid, comfortable anaesthesia for diagnostics and brief surgeries [35,39]. Tetracaine’s duration suits PRK [40], while lidocaine gel benefits paediatric cases [41,42]. Emerging formulations combining proxymetacaine with adjuvants like TTX may optimise duration and safety [36,45]. Clinicians must balance procedure needs with patient risks, particularly regarding corneal healing and comorbidities [31,37,44].

The cornea is thought to contain 300–400 times more small nerve fibres than human skin. The pain mechanisms following epi-off CXL remain inadequately understood [46]. Research on factors influencing post-eye surgery pain reveals a lack of studies in this area [47]. Henzler et al. have demonstrated that the intensity of postoperative pain is most significant within the initial six hours following any ophthalmic procedure [48]. Predictive factors for pain, such as age and sex, were investigated in many studies, and a correlation with age was observed in most of them [49]. However, concerning sex, it was investigated in fewer studies, and the conclusions of these studies were inconsistent [49]. We did not find any relationship between age or sex and pain and healing response in our study. However, the age range in our study was considerably narrow, as progressive keratoconus patients are usually young.

Anxiety has been recognised as a significant predictor of postoperative pain, particularly in the context of obstetrical, gynaecological, and gastrointestinal procedures [49]. Anxiety has been linked to increased pain sensitivity, supporting the concept that emotional states regulate pain reactivity in humans [49,50]. Another essential determinant of intrapersonal aspects of pain, such as increased pain intensity, distress, and disability, is pain catastrophizing [51]. Although incompletely understood, the factors underlying individual differences in pain response likely include personality type [52].

Compared to other corneal procedures, post-CXL pain is transient and does not typically progress to a chronic state. Some procedures, such as laser-assisted in situ keratomileusis (LASIK), can exhibit delayed pain, which may manifest between one and twenty-four months following the procedures [53]. It is well known that corneal erosion induces excruciating pain on its own. Nevertheless, pain intensity appears more pronounced following crosslinking than photorefractive keratectomy. This finding suggests there are additional variables that induce pain associated with the CXL treatment [18]. As previously hypothesised, UVA-generated free radicals may also contribute to lipid peroxidation and prostaglandin production in CXL [19]. Epi-on or transepithelial CXL studies have demonstrated that epi-on CXL reduces both the intensity and duration of pain experienced by patients. The observed discrepancy may be attributed to the lack of de-epithelialization and corneal nerve injury. An additional benefit of epi-on CXL is the reduction in risk for complications such as corneal opacity and infectious keratitis while also expediting postoperative recovery [15,54]. However, the overall efficacy of epi-on CXL is less effective in halting the progression of keratoconus than epi-off CXL [3,4]. Typical pharmacological management of postoperative pain involves the application of local anaesthetic eye drops during the procedure, followed by antibiotic eye drops and a topical steroid to reduce inflammation. Various oral analgesics, including paracetamol, nonsteroidal anti-inflammatory medicines (NSAIDs), gabapentin, and oxycontin, have been employed as pain relief medications. However, comprehensive evaluations of the most effective medications and dosages for this purpose have yet to be established in the literature. Gabapentin and nonsteroidal anti-inflammatory drugs (NSAIDs) are used postoperatively in corneal refractive procedures and have the potential to alleviate pain associated with CXL. In contrast to ketorolac, which inhibits inflammation, gabapentin functions as an analogue of an inhibitory neurotransmitter. The effectiveness of gabapentin and ketorolac in managing pain after CXL is comparable in both epi-off and epi-on techniques [21]. It was reported that using bandage contact lenses does not significantly impact pain scores compared to alternative postoperative protocols such as occlusive patching or ointment [22]. It is also reported that bandage contact lens application can result in significantly faster healing times, reduced pain levels, and smaller epithelial defect sizes compared to a pressure patching group [28,55,56]. As bandage contact lenses are applied to all epithelium-off CXL patients in our department as per our local protocol, it is unlikely to influence our primary outcome. However, future studies looking into the separate influence of bandage contact lenses and proxymetacaine would be useful. Wearing the lenses increases the risk of developing microbial keratitis, and bacterial proliferation on bandage contact lenses has been documented in nearly 30% of patients, according to one study [57]. Therefore, prophylactic use of topical antibiotics is necessary when using bandage contact lenses.

Optimal pain management and its potential benefits are greatly needed not when the perioperative local anaesthesia subsides but in the middle of the first postoperative night. A feasible approach could involve administering a low-dose local anaesthetic to the operated cornea via extended release overnight. This could be achieved using a bandage contact lens drug delivery system [58]. Following photorefractive keratectomy (PRK), a diluted topical anaesthetic exerts a direct analgesic effect. Nevertheless, since the earliest PRK cases, this practice has been questioned and often avoided due to its harmful impact on keratocytes and the cornea epithelium [59,60]. Studies have demonstrated that reduced concentrations of topical anaesthetic drops, including proparacaine and bupivacaine, do not induce stromal injury or postpone corneal re-epithelialization following PRK surgery [60,61,62]. Verma et al. [62] applied local anaesthetic eye drops containing 1% preservative-free tetracaine hydrochloride every 30 min during twenty-four hours of waking hours with adequate pain control results. Additionally, they observed that the topographic irregularity identified at the one-week follow-up in two placebo-treated patients and five tetracaine-treated patients may have been caused by a combination of the frequent instillation of drops and lid movement. However, the topography of all patients remained normal at the one-month follow-up in their study.

Our sole reliance on proxymetacaine to manage pain was because it can be readily prescribed to patients in the National Health Service in the UK and induces less distress and toxicity when given as opposed to oxybupracaine or tetracaine. However, all three were demonstrated to be effective and safe, with only rare instances of corneal toxicity observed when applied within prescribed limits and not abused; they did not extend the period of recovery of the corneal epithelium [63,64,65]. In the early postoperative period, Brilakis et al. [65] provided only ten drops of tetracaine to each patient to use as required to control the risk of anaesthetic abuse. There is still considerable room for innovation regarding methods to assist patients in managing postoperative pain [66]. While cases of topical anaesthetic toxicity have been reported due to prolonged and unsupervised use, as discussed above [30,31], our structured protocol was designed to mitigate these risks. Patients were provided with a limited quantity of proxymetacaine (20 single-use minims, Bausch & Lomb, USA), instructed to use the drops as minimally and possible and up to a maximum of eight times daily for 3 days, and were carefully explained the risks of intensive use. They were also instructed to contact us if the pain lasted more than 3 days. This controlled short-term regimen, along with early follow-up and careful monitoring, reduced the potential for abuse and epithelial toxicity. Proxymetacaine hydrochloride 0.5% exemplifies the delicate balance between therapeutic benefit and iatrogenic risk inherent to potent topical anaesthetics, demanding vigilant clinical oversight to prevent misuse while harnessing its unique pharmacodynamic properties for optimal patient care.

The main limitation of this study is its single-centre retrospective observational design without a control group, and the study did not utilise a validated pain scale measurement, as the primary aim was to subjectively assess the number of patients complaining of pain after 3 days We acknowledge that the absence of a control group limits the ability to fully attribute the observed pain relief solely to the use of proxymetacaine hydrochloride but as discussed earlier bandage contact lenses does not significantly impact pain scores compared to alternative postoperative protocols in previous studies. Another limitation was that our normal post-CXL protocol did not include any routine imaging at first follow-up; we relied only on slit-lamp examination with fluorescein staining but did not utilise adjunctive imaging modalities for epithelial evaluation. Further prospective, randomised studies comparing the use of proxymetacaine with a control group not using proxymetacaine, examining pain in relation to epithelial healing, are necessary to isolate the specific analgesic effect of proxymetacaine and to better understand its role in postoperative pain management after epi-off CXL Nevertheless, our pain management protocol has consistently yielded positive outcomes for a decade when implemented in a large number of patients. This inspired us to disseminate our findings, providing others with evidence that could potentially influence their practices and motivating other researchers to further investigate this domain by conducting prospective case–control trials examining the efficacy of topical anaesthetics as a prescribed pain control medication.

In summary, patients consistently did not express subjective postoperative pain after 3 days, with no adverse effects if used restrictively (up to eight times a day in the first three days only) after epi-off CXL.

## Figures and Tables

**Table 1:** Crosslinking protocol.

	Non-Accelerated CXL (Brighton, UK)
**Treatment target**	Progressive keratoconus
**Progression criteria**	>1 D increase in K max over 6–12 months
**Fluence (J/** **cm^2^** **)**	5.4
**Soak time and interval**	10 min, q1
**Intensity (mW)**	3
**Treatment time (minutes)**	30 min
**Epithelium status**	Epithelium-off
**Chromophore**	Riboflavin (Vibex Rapid, Avedro, USA)
**Chromophore carrier**	HPMC
**Chromophore osmolarity**	Iso-osmolar
**Chromophore concentration**	0.1%
**Light source (device name)**	Avedro (Avedro KXL system, Waltham, MA, USA)
**Irradiation mode (interval)**	Continuous
**Protocol modifications**	nil
**Protocol abbreviation in manuscript**	Epi-off CXL

## Data Availability

The data presented in this study are available on request from the corresponding author.
